# Multivariate Analyses with Two-Step Dimension Reduction for an Association Study Between ^11^C-Pittsburgh Compound B and Magnetic Resonance Imaging in Alzheimer’s Disease

**DOI:** 10.3390/bioengineering12010048

**Published:** 2025-01-09

**Authors:** Atsushi Kawaguchi, Fumio Yamashita

**Affiliations:** 1 Faculty of Medicine, Saga University, 5-1-1 Nabeshima, Saga 849-8501, Japan; 2 Division of Ultrahigh Field MRI, Iwate Medical University, 1-1-1 Idaidori, Yahaba 028-3694, Japan

**Keywords:** Alzheimer’s disease, PiB imaging, magnetic resonance imaging, matrix decomposition, multimodal

## Abstract

The neuropathological diagnosis of Alzheimer’s disease (AD) relies on amyloid beta (Aβ) deposition in brain tissues. To study the relationship between Aβ deposition and brain structure, as determined using ^11^C-Pittsburgh compound B (PiB) and magnetic resonance imaging (MRI), respectively, we developed a regression model with PiB and MRI data as the predictor and response variables, respectively, and proposed a regression method for studying the association between them based on a supervised sparse multivariate analysis with dimension reduction based on a composite paired basis function. By applying this method to imaging data of 61 patients with AD (age: 55–85), the first component showed the strongest correlation with the composite score, owing to the supervised feature. The spatial pattern included the hippocampal and parahippocampal regions for MRI. The peak value was observed in the posterior cingulate and precuneus for PiB. The differences in PiB scores among the diagnosis groups 12 months after PiB imaging were significant between the normal and AD groups (*p* = 0.0284), but not between the normal and mild cognitive impairment (MCI) groups or the MCI and AD groups (*p* = 0.3508). Our method may facilitate the development of a dementia biomarker from brain imaging data. Scoring imaging data allows for visualization and the application of traditional analysis, facilitating clinical analysis for better interpretation of results.

## 1. Introduction

Alzheimer disease (AD) is the most common form of dementia, and its incidence is growing worldwide [1]. The neuropathological diagnosis of AD relies on the deposition of amyloid beta (Aβ) in the brain tissue. The spatial pattern of Aβ deposition can be visualized by administering the radiotracer ^11^C-Pittsburgh compound B (PiB) and performing subsequent positron emission tomography (PET) [2]. When performing regression analysis, response variables based on cognitive function tests are often used. Chapleaut et al. [3] provide a comprehensive review of the latest applications of amyloid PET in neurodegenerative diseases. Amyloid PET is important in the diagnosis of AD and can minimally invasively detect amyloid plaques. With the approval of anti-amyloid monoclonal antibodies, their use has increased, helping treat mild cognitive impairment (MCI) and mild dementia. It has also been applied to neurodegenerative diseases other than AD. In particular, it has been applied to the study of dementia with Lewy bodies (DLB), Parkinson’s disease dementia (PDD), and Parkinson’s disease (PD) and some specific studies are presented below. The study by Edison et al. [4] measured amyloid load in DLB and PDD patients using ^11^C-PiB PET; they observed a marked increase in amyloid load in approximately 80% of DLB patients, while PDD patients showed less of an increase in amyloid. A study by Donaghy et al. [5] evaluated the utility of amyloid PET in DLB. In particular, DLB patients showed a greater accumulation of amyloid plaques than DLB patients. In particular, we found that DLB patients often had less amyloid accumulation; DLB patients tended to have higher Aβ ligand binding than PDD patients. PD without dementia does not show increased binding. The Walker et al. [6] study examined the prevalence, distribution, and severity of cerebral amyloid angiopathy (CAA) in DLB, PDD, and PD, showing that the distribution and severity of CAA in DLB is similar to that in AD. This may be related to the development of dementia. Amyloid PET images such as PiB image are interpreted visually and evaluated using quantification methods such as SUV ratios. It is also important in clinical trials and utility studies, where it is compared with other biomarkers and analyzed in detail. This approach is less informative because the diagnosis is a univariate, categorical variable. Indeed, cognitive function tests play an important role in the diagnosis of AD, such as the Mini-Mental State Examination (MMSE), a simple cognitive assessment tool that evaluates memory, attention, computation, language, and visuospatial cognition, the Montreal Cognitive Assessment, which provides a more detailed assessment than MMSE and is particularly superior in detecting MCI, and the clock-drawing test, a simple test that assesses visuospatial cognition and executive function. These tests may include information directly related to the illness, as well as other factors. This is because of the wide range of difficulties of the questions and their content. Additionally, patients’ cultural backgrounds and educational levels may influence test results. Thus, in this study, images that can assess pathophysiology more directly than cognitive test functions are used as the objective variable.

Magnetic resonance imaging (MRI) is a useful tool for examining the structural changes caused by Aβ deposition in the brain and provides more relevant response information than cognitive tests. This concept is especially useful in imaging genetics [3]. In this context, brain imaging data were used to identify the intermediate phenotypes. Multiple datasets have been classified into multimodal imaging analyses in neuroinformatics research and multiomics in bioinformatics research, which are among the most widely explored topics. These are being advanced through the development of new methodologies and technologies and have the potential for major breakthroughs in the understanding of human health and disease [7]. Consequently, the use of structural MRI (sMRI) as a response is expected to yield more information through regression analysis. However, sMRI provides high-dimensional data with millions of voxels as predictor variables, making statistical analysis very challenging unless dimensionality reduction is performed.

Generally, in multivariate analyses of brain imaging using response and predictor variables, partial least squares (PLS), canonical component analysis (CCA), reduced rank regression, and parallel independent component analysis (ICA) have been used. These approaches are common because the datasets are scored using a linear combination but differ in the objective function used to compute weights. In brain imaging analyses, methods other than parallel ICA can be considered equivariant [8]. Although parallel ICA offers high decomposability, its incorporation of sparsity is challenging, leading to increased use of other methods. Lin et al. [9] showed that sparse models are effective in the integrated analysis of high-dimensional and heterogeneous imaging and genetic data. In the present study, an extension of the PLS or CCA was considered. Including imaging data as a response variable presents challenges owing to its large dimensionality and high correlations. Because voxel-based imaging data serve as both predictors and responses in the model, a multivariate approach with dimensionality reduction would be ideal. Although PLS and CCA can deal with correlated data and reduce dimensions, they may lead to the loss of important information, such as spatial relationships. Moreover, the objective function for weights maximizes the correlation between scores, and the resulting scores are not always related to the outcomes. Kawaguchi and Yamashita [10] developed a supervised and multiblock version of the PLS or CCA to analyze multimodal imaging and genetic data. In this study, we aimed to evaluate the relationship between Aβ deposition and brain structure, as determined using PiB and MRI. Moreover, we identified the optimal brain regions for assessing this relationship. A regression model was used with PiB data as the predictor and MRI data as the response variable. Furthermore, we propose a regression method for studying the association between PIB and MRI based on a supervised sparse multivariate analysis (SSMA) method with dimension reduction based on a composite paired basis function. This is a modified method of that proposed by Yoshida et al. [11] (radial basis function-sparse PLS without supervision and with a single basis function) and that of Kawaguchi and Yamashita [10] (multiblock sparse multivariable analysis). Initially, spatial brain images are reduced in dimensionality via basis expansion, followed by further dimensionality reduction using regularized matrix factorization. This is supervised by a univariate composite score representing a linear combination of cognitive test scores, enabling the simultaneous data-driven selection of relevant brain regions. While previous studies take the means of condensing to anatomical regions due to the huge voxel values, the proposed method is able to capture voxel-level relationships among brain images, and furthermore, super vision improves interpretability. These are considered to be the main advantages of the proposed method. Because it is a flexible method that is not restricted to specific information, we believe it has the potential to be applicable to a wider variety of clinical applications.

## 2. Materials and Methods

### 2.1. Data

This method was applied to real data from the AD Neuroimaging Initiative, which is a collection of imaging data from 61 individuals aged 55–85. We used MRI data obtained 12 months after PiB imaging.

### 2.2. Preprocessing

For image preprocessing, three-dimensional (3D) T1-weighted images of the participants were first segmented into gray matter (GM), white matter, and cerebrospinal fluid space, followed by anatomical normalization to a template image using DARTEL [12]. For PiB, images were coregistered to the bias-corrected T1 image, and the same warping parameter as that for T1 images was applied following intensity normalization to the cerebellar GM for each participant.

### 2.3. Notation

$S_{Y}$ and $S_{X}$ are the n×N matrices for PiB and sMRI data, respectively. Each row corresponds to vectorized 3D imaging data for each participant.

### 2.4. Dimension Reduction

In the first phase, the number of dimensions for the imaging data are reduced by applying basis expansion. Since sMRI and PiB data have the same dimensions, we apply a common basis function to reduce the number of parameters from N to q. $Y=S_{Y}B$ and $X=S_{X}B$ are the n×q matrix, where $S_{m}=\left\{s_{m\alpha }\right\}$ is an n×N matrix, and $B={\left\{\phi_{k}\left(v_{j}\right)\right\}}_{j=1,\ldots ,N,k=1,\ldots ,q}$ is an N×q matrix. Each element in **B** is defined by a radial B-spline function, which provides a basis for reducing the dimensions of the original data.

$$\phi_{k}\left(w\right)=\frac{1}{4h^{2}}\left\{\begin{array}{c} h^{3}-3h^{2}d_{k}\left(w\right)+3h{d_{k}\left(w\right)}^{2}+3{d_{k}\left(w\right)}^{3}\\ {\left(h-d_{k}\left(w\right)\right)}^{3}\\ 0 \end{array}\begin{array}{c} \left(d_{k}\left(w\right)\leq 0\right)\\ \left(0<d_{k}\left(w\right)\leq h\right)\\ \left(d_{k}\left(w\right)>h\right) \end{array}\right.,$$

where $d_{k}\left(w\right)=∥w-\kappa_{i}∥-h$, $\kappa_{i}\in Z^{3}$ is the pre-specified knot, h>0 is the distance between knots, and $\left\|a\right\|=\sqrt{a_{1}^{2}+a_{2}^{2}+a_{3}^{2}}$ for 3D vector a. Notably, basis expansion was useful for 3D neuroimaging analysis, as also reported by Araki et al. [13,14], Yoshida et al. [11,15], Kawaguchi and Yamashita [10], and Kawaguchi [16].

The number of basis functions, q, is determined by the number of voxels and the distance between the prespecified knots. In this study, we employed four-voxel equal-spacing knots, resulting in a distance $h=\sqrt{3\times 4^{2}}=6.93$. This choice was influenced by our previous simulation study [8], which indicated that a smaller distance between knots could improve accuracy.

**Figure 1:**
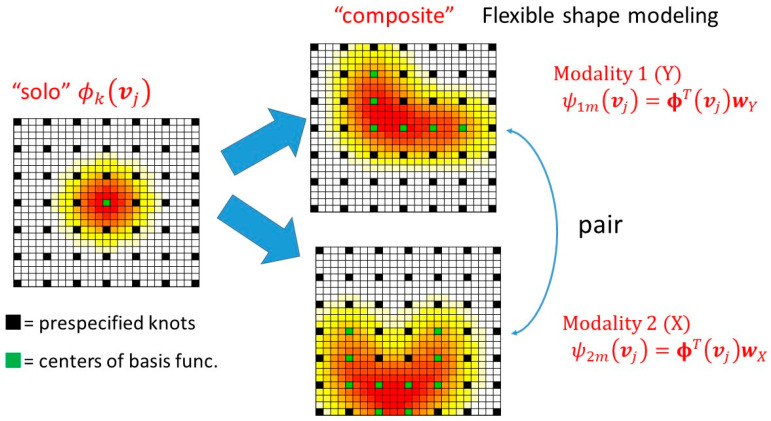
Composite paired basis functions. The heat color represents the shape of the function.

Dimension reduction based on the basis function was followed by the application of the SSMA method. Thus, the result is obtained as a linear combination of the basis functions $\psi_{X}\left(v_{j}\right)=\sum_{k=1}^{q}\phi_{k}\left(v_{j}\right)w_{X,m}$ and $\psi_{Y}\left(v_{j}\right)=\sum_{k=1}^{q}\phi_{k}\left(v_{j}\right)w_{Y,m}$, which is referred to as a composite function (Figure 1). $\psi_{X}\left(\cdot \right)$ and $\psi_{Y}\left(\cdot \right)$ are paired because of the hierarchical structure. This can be regarded as a paired composite function, which represents related brain regions across modalities. The composite basis $\psi \left(v\right)$ can exhibit a flexible shape, whereas the original basis function $\phi_{k}\left(v\right)$ maintains a spherical shape.

### 2.5. Supervised Sparse Multivariate Analysis

In this section, we present the proposed SSMA method. Consider n subject, with X as the n×p predictor matrix and Y as the n×q response matrix. Each subject also has a univariate outcome represented by the n-dimensional vector Z. We define scores t for the predictor and u for the response, with the following structure:

(1) $$t=Xw_{X},\;u=Yw_{Y},$$

where $w_{X}$ and $w_{Y}$ are the weight vectors for X and Y, respectively. Subsequently, matrices X and Y are normalized by their columns. The association between the two datasets (images) was evaluated by maximizing the correlation between the super scores t and u supervised by the outcomes. Weights $w_{X}$ and $w_{Y}$ were estimated by maximizing the following function:

(2) $$L\left(w_{X},w_{Y}\right)=\mu_{XY}t^{⊤}u+\mu_{XZ}t^{⊤}Z+\mu_{YZ}u^{⊤}Z-P_{\lambda_{X}}\left(w_{X}\right)-P_{\lambda_{Y}}\left(w_{Y}\right)$$

$subject\;to\;\|w_{X}{\|}^{2}=1\;and\;\|w_{Y}{\|}^{2}=1$, where $0\leq \mu_{XY},\mu_{XZ},\mu_{YZ}\leq 1$ and $\mu_{XY}+\mu_{XZ}+\mu_{YZ}=1$ are proportions of the supervision. The penalty function $P_{\lambda }\left(x\right)$ is defined as $P_{\lambda }\left(x\right)=2\lambda \left|x\right|$ in this paper, where λ>0 is the regularization parameter that controls sparsity, thereby facilitating the detection of associated regions in the brain or genome. It is possible for the penalty function to have other structures; however, these structures are beyond the scope of this study. When the proportions of supervision for the predictor-response pairs ($\mu_{XZ}$ and $\mu_{YZ}$) are both set to zero, the proposed method simplifies to either multiblock PLS or CCA.

We estimate the weights in (1) by stating the following proposition.

**Proposition 1** **(coordinate updates).** *The solution of optimization problem (2) satisfies*

$${\tilde{w}}_{X}=h_{\lambda_{X}}\left(X^{⊤}\left\{\mu_{XY}u+\mu_{XZ}Z\right\}\right),\;{\tilde{w}}_{Y}=h_{\lambda_{Y}}\left(\left\{\mu_{XY}t^{⊤}+\mu_{YZ}Z^{⊤}\right\}Y\right)$$

*where* $h_{\lambda }\left(y\right)=sign\left(y\right){\left(\left|y\right|-\lambda \right)}_{+}$ *is the sparse function.*

The proof can be obtained in a manner similar to that reported by Kawaguchi and Yamashita [10]. This leads to the following algorithm for estimating the weights in (1) by maximizing L in (2).

1. Initialize t and u and normalize the super scores as follows:
  $$t\leftarrow t/{\left\|t\right\|}_{2},\;u\leftarrow u/{\left\|u\right\|}_{2}$$
  where ← means “replaced with”.
2. Repeat until convergence.
  2.1 For fixed u, ${\tilde{w}}_{X}=h_{\lambda_{X}}\left(X^{⊤}\left\{\mu_{XY}u+\mu_{XZ}Z\right\}\right)$ and normalize ${\hat{w}}_{X}$ = ${\tilde{w}}_{X}/{\left\|{\tilde{w}}_{X}\right\|}_{2}$.
  2.2 Putting $t=X{\hat{w}}_{X}$, ${\tilde{w}}_{Y}=h_{\lambda_{Y}}\left(b_{Y}\left\{\mu_{XY}t^{⊤}+\mu_{YZ}Z^{⊤}\right\}Y\right)$ then normalize ${\hat{w}}_{Y}$ = ${\tilde{w}}_{Y}/{\left\|{\tilde{w}}_{Y}\right\|}_{2}$.
  2.3 Set $u=Y{\hat{w}}_{Y}$,
3. Set $p=X^{⊤}t/t^{⊤}t$ and $q=Y^{⊤}u/u^{⊤}u$, $X\leftarrow X-tp^{⊤}$ and $Y\leftarrow Y-uq^{⊤}.$

Note that by iterating Steps 1–3, the deflation step (Step 3) yields multiple components (k=1,2,…,K), the new X and Y obtained in Step 3. The convergence of this algorithm is supported by the proposition provided by Kawaguchi and Yamashita [10]. The method for selecting the regularization parameter was consistent with the approach reported by Kawaguchi and Yamashita [10], which is based on the information criterion. The R package “msma” was used to implement the method described in this section and is available from the Comprehensive R Archive Network at http://CRAN.R-project.org/package=msma (accessed on 8 November 2024).

### 2.6. Statistical Analysis

The relationships between sMRI and PiB scores estimated using SSMA were evaluated using Pearson’s correlation coefficients. The scores are calculated as standardized continuous values and outliers are rarely observed. Therefore, Pearson’s correlation coefficient is considered to be applicable. The resulting PiB scores in the SSMA were tested using Tukey’s multiple comparison test. This validation was performed by using non-imaging data of future diagnoses (normal, MCI, and AD groups) and comparing them between groups. The comparison was limited to cases with diagnostic information. Statistical significance was set at *p* < 0.05.

### 2.7. Simulation Study Setting

In this section, the characteristics of the proposed method are presented based on synthetic data. Specifically, the aim is to demonstrate how the supervised feature functions work, mainly by comparing the original method with the effects of varying $\mu_{YZ}$ and $\mu_{XZ}$. Notably, as described in Step 2, the case of $\mu_{XZ}=\mu_{YZ}=0$ corresponds to the original method (unsupervised version).

**Figure 2:**
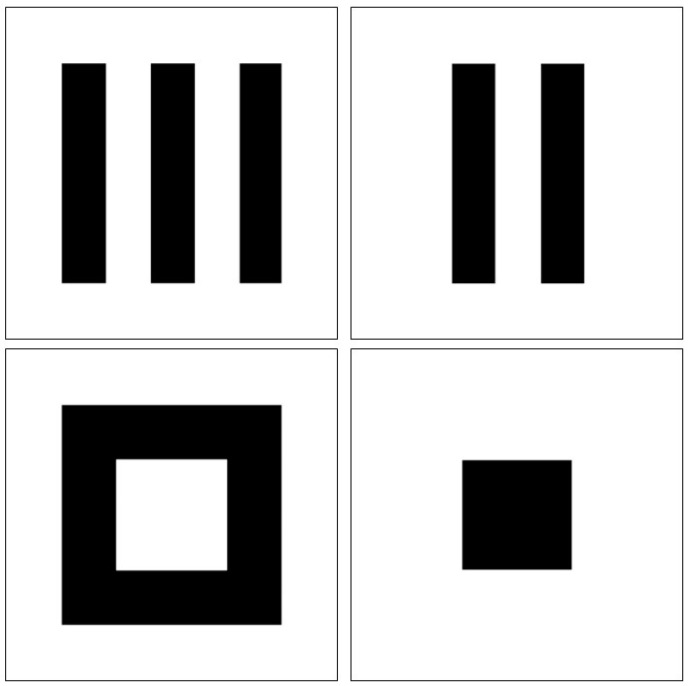
True images for the simulation study.

We considered a sample size of n=50 subjects and set the number of components to K=2. The true images were 2D with 100×100 pixels (q=10,000), as shown in Figure 2. The observed data consisted of $\left(X,Y,Z\right),$ where X is the predictor matrix, Y is the response matrix, and Z is the outcome vector. Both X and Y are n×10,000 matrices generated from the n×K (latent) scores t and u, respectively, with the true images serving as weights. The number of columns in t and u corresponds to the number of components. The outcome measured by Z is influenced by the latent factors represented in u.

First, t is obtained by generating a multivariate normal random number from $N\left(0,I_{2}\right)$. Subsequently, u is generated from the relationship between t and u, represented as

$$u=tw_{u}^{⊤}+\varepsilon ,$$

where $w_{u}={\left(1,w_{u,0}\right)}^{⊤}$, $w_{u,0}=0.2\;or\;0.8$, and $\varepsilon ∼N\left(0,I_{2}\right)$. In the second component, the relationships between scores are considered to be either weak or strong.

The predictor and response matrices were generated as follows. The predictor is given by

$$X=tw_{X}^{-}$$

where $x^{-}$ is the generalized inverse for x. The weight for images was $w_{X}=s_{X}w_{X}$ with the k-th column of $s_{X}$ as $s_{X,k}={\left(s_{X,k}\left(v_{1}\right),s_{X,k}\left(v_{2}\right),\ldots ,s_{X,k}\left(v_{q}\right)\right)}^{⊤}$ is the vectorized true images and $w_{X}=$ 5. The response is given by

$$Y=uw_{Y}^{-}+E$$

where $w_{Y}=s_{Y}w_{Y}$ with the k-th column of $s_{Y}$ as $s_{Y,k}={\left(s_{Y,k}\left(v_{1}\right),s_{Y,k}\left(v_{2}\right),\ldots ,s_{Y,k}\left(v_{q}\right)\right)}^{⊤}$ is the vectorized true images, $E=e_{0}E_{0}$ by considering the noise level $e_{0}=0.005$ and n×q matrix $E_{0}$ with (i,j)-th element $e_{0,i,j}∼N\left(0,1\right)$. The outcome is given by $Z=uw_{z}^{⊤}$ with $w_{z}={\left(1,0\right)}^{⊤}$; briefly, only the first component is related with the outcome.

Thus, the case $w_{Y}=1$ represents a strong effect of the score on the observed data, and the case $w_{Y}=20$ ($w_{Y}^{-1}=0.05$) represents a weak effect. Thus, only the Y-score for the second component is associated with the outcome.

The SSMA method is applied to observed data $\left(X,Y,Z\right)$ with parameter combinations with $\mu_{XZ}=0,0.25,0.5,0.75$ and $\mu_{YZ}=0,0.25,0.5,0.75$ satisfying $\mu_{XZ}+\mu_{YZ}\leq 1$ by using two components. The iterations for generating data and applying the method were conducted 100 times for each parameter pair. The radial B-spline function with 2-pixel equal spacing knots is used.

## 3. Results

### 3.1. Application

**Figure 3:**
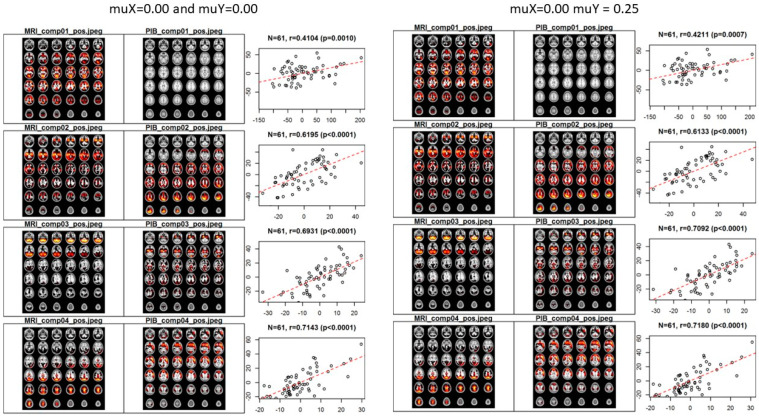
Spatial pattern results in the SSMA model.

Figure 3 illustrates the spatial pattern and correlation among scores by components for cases $\left(\mu_{XZ},\;\mu_{YZ}\right)=\left(0,0\right)$ and $\left(\mu_{XZ},\;\mu_{YZ}\right)=\left(0,0.25\right)$. Four components were selected for both cases. The first component exhibited the highest correlation with a composite score, influenced by the supervised nature of the analysis. In the second component, a relationship that makes sense from a biological perspective was found, suggesting that the data reflect known anatomical or functional relationships in the brain. The spatial pattern included the hippocampal and parahippocampal regions for MRI, while peak values for PiB data were observed in the posterior cingulate and precuneus. These are considered reasonable areas based on previous studies, and further description will be given in the discussion section. For all components, except for the second one in the case of $\left(\mu_{XZ},\;\mu_{YZ}\right)=\left(0,0.25\right),$ the relationships among scores were stronger than those observed in the case of $\left(\mu_{XZ},\;\mu_{YZ}\right)=\left(0,0\right)$.

**Figure 4:**
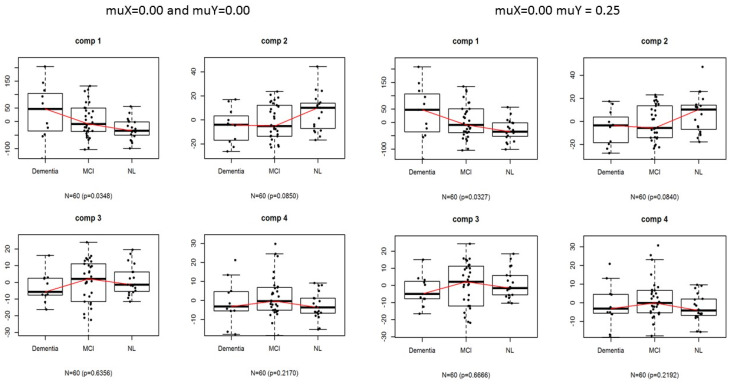
PiB scores in the SSMA model.

The differences in PiB scores among the diagnosis groups 12 months after PiB imaging are presented in Figure 4. When comparing the three groups—normal, MCI, and AD—statistical significance was observed only in the first component of the analysis. This means that the differences among these groups were notable in that specific component. In contrast, in the second component, a significance level of approximately 10% was noted, which suggests that the differences among the groups were less pronounced or only marginally significant in this context. The differences in the PiB first component scores among the diagnosis groups 12 months after PiB imaging were significant between the normal and AD groups (*p* = 0.0284), but not between the normal and MCI groups or the MCI and AD groups (*p* = 0.3508).

### 3.2. Simulation Study

All resulting weights from the SSMA method were binarized, categorizing weights as either zero (being equal, unselected) or non-zero (selected). The c-index was then computed using the true images and variables. The c-index is defined as

c-index = sensitivity − (1 − specificity).

**Figure 5:**
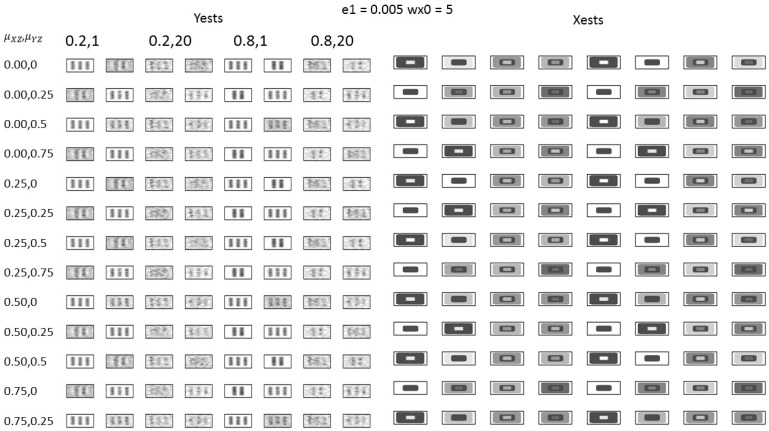
Probability 2D maps for the result of the simulation study.

The average values of the c-index were derived from 100 simulations, using various parameter combinations, specifically $\left(w_{u,0},w_{Y}\right)=\left(0.2,20\right),\;\left(0.8,\;1\right),$ along with $\mu_{XZ}$, and $\mu_{YZ}$. These parameters influence how the model behaves and the results it generates. The results are summarized in Table 1 and Table 2 for $e_{0}=0.005$. Notably, to facilitate interpretation, $w_{Y}$ is displayed as the inverse $w_{Y}^{-1}$. The results were divided into X and Y, with the c-index for each component listed. The average values from the two components are presented in the third and sixth columns, while the last column displays the average value for both X and Y across the two components. The highest mean value among $\mu_{XZ}$ and $\mu_{YZ}$ for each $w_{u,0}$ and $w_{Y}$ is highlighted in bold. Figure 5 illustrates the 2D displays of the resulting probabilities for Y and X for $e_{0}=0.005$. These probabilities are computed from the pixel-wise mean value of the binary images, indicating whether a pixel is selected in the estimation.

In general, the supervised feature with $\mu_{XZ}>0$ or $\mu_{YZ}>0$ consistently resulted in better predictive accuracy, as evidenced by higher c-index values, than the unsupervised cases with $\mu_{XZ}=0$ and $\mu_{YZ}=0$.

In the case of $w_{u,0}=$ 0.2, which represents a lower association between the scores u and t of X and Y, respectively, the difference in $\mu_{YZ}$ may not be significant. Conversely, when $w_{u,0}=0.8$, which indicates a higher association between the scores of X and Y, the supervised method with $\mu_{YZ}>0$ demonstrated superior performance to the original method with $\mu_{YZ}=0$. Furthermore, in the case of $w_{Y}=$ 1, the supervised method with $\mu_{XZ}+\mu_{YZ}<1$ outperformed the original method with $\mu_{XZ}+\mu_{YZ}=1$. In contrast, when $w_{Y}=20$, the supervised method with $\mu_{XZ}+\mu_{YZ}=1$ outperformed the original method with $\mu_{XZ}+\mu_{YZ}>1$.

In the case of $w_{Y}=20$, detecting the true images in Figure 5 was challenging. In contrast, for $w_{Y}=1$, the true images were successfully identified. The best performance was observed for $\left(\mu_{XZ},\;\mu_{YZ}\right)=\left(0.25,0.5\right)$. This was mainly owing to the data structure of the simulation, where only the second component was associated with the outcome. However, the over-supervised ($\mu_{XZ}+\mu_{YZ}=1$) resulted in poor reconstruction of the true image. Other parameter-setting patterns ($\left(w_{u,0},w_{Y}\right)=\left(0.2,1\right),\;\left(0.8,\;20\right)$) are presented in Table A1 and Table A2 of Appendix A, and the findings are consistent with the previous results.

## 4. Discussion

This paper presents a novel method for analyzing high-dimensional datasets. In practical applications, the scores calculated by the proposed method allow the use of traditional statistical analysis methods such as analysis of variance, which is usually used in clinical data analysis. It is also possible to perform logistic regression analysis and calculate the probability of disease diagnosis according to the value of the score. From a methodological perspective, we developed an SSMA method that can determine the association between multivariable datasets incorporating clinical outcomes. From an application perspective, we developed a method to predict dementia from brain images and to identify associations with other brain images using the following key features: (1) a paired composite basis function with a data-driven shape, (2) data-driven brain regions, and (3) availability for brain-wide analysis. Many studies focus on the regions specified by previous studies before analyzing them. For example, in studies on AD, the hippocampal region may be focused on. Also, in analyses of the whole brain, anatomically segmented regions of interests such as the representative Automated Anatomical Labelling Atlas may be used. In contrast, the proposed method does not require a predefined segmentation for analysis on a voxel-by-voxel basis, and thus can be flexibly applied to other diseases. In this study, we adopt an application in well-studied AD that also allows the evaluation of regions. These features of our method allow us to derive associations that are easier to interpret and can be applied to other medical big data analyses.

CCA and PLS are traditional methods that are highly effective for regression analysis of multivariate data. In this study, we extend this method to facilitate regression analysis involving both explanatory and objective variables represented as image data. While CCA and PLS are dimensionality reduction methods, further dimensionality reduction is required to analyze images with large amounts of information. The final result achieved dimensionality reduction using a flexible representation of the brain region. Additionally, we incorporated disease-related variables beyond imaging data as “teacher” data during parameter estimation to obtain results that better reflect the pathological condition. Simulation studies and real data analyses demonstrated the usefulness of this method. The choice of teacher data is determined on a study-by-study basis, and the results may vary depending on this choice.

The spatial patterns identified in this study highlighted the hippocampal and parahippocampal regions as significant spatial patterns on MRI. In contrast, PiB-PET scans frequently revealed peak values in the posterior cingulate gyrus and precuneus. These regions are essential indicators for early diagnosis and progression assessment of AD. Yokoi et al. [17] used PiB-PET and THK5351-PET to examine the relationship between Aβ accumulation and functional networks in the posterior cingulate gyrus and precuneus of patients with AD. Results indicate that Aβ accumulation in these regions is strongly associated with cognitive decline. For instance, a meta-analysis by Ballmaier et al. [18] used MRI to assess atrophy of the hippocampus and posterior cingulate gyrus in patients with AD, demonstrating marked atrophy associated with the disease. Teipel et al. [19] further reported hypometabolism and hypoperfusion of the posterior cingulate gyrus and precuneus in MCI and AD. Additionally, Storandt et al. [20] employed PiB-PET scans to investigate the association between Aβaccumulation, cognitive decline, and brain volume loss, supporting the link between these pathological features and disease progression. Based on this, early detection of Aβ accumulation will allow for early intervention to slow the progression of AD. Specific treatment recommendations include anti-amyloid medications (e.g., aducanumab) and lifestyle modifications. A systematic review by Teipel et al. [19] evaluated different neuroimaging correlates in patients with MCI and AD. Hypometabolism and hypoperfusion of the posterior cingulate gyrus and precuneus emerged as key indicators for MCI and AD, underscoring these regions’ role in disease progression.

This study has some limitations. The model’s performance relies on the choice of “teacher” data during parameter estimation. As different studies may select different clinical variables as “teacher” data, results may vary, potentially limiting reproducibility and generalizability across studies with different data sources. Additionally, this study primarily focuses on data associated with AD. Expanding validation across other neurodegenerative conditions or broader, non-pathological datasets could reveal limitations or areas where the model may not generalize well. Future studies could investigate criteria for selecting “teacher” data, optimizing variable choices to enhance model robustness and transferability across different datasets and study designs. Furthermore, additional imaging modalities beyond MRI and PET, such as functional MRI, diffusion tensor imaging, or quantitative susceptibility mapping, could be incorporated by extending the proposed method to multi-block methods, improving the detection of biomarkers across a broader spectrum of brain function and structure. These verification studies will be the subject of future research.

The proposed method facilitates constructing a prediction model that uses images as the response variable, offering greater predictive objectives than other univariate variables, such as cognitive test results. This approach is expected to yield novel insights through exploratory analysis.

## 5. Conclusions

The reported method for studying the association between the two imaging modalities will facilitate the development of a biomarker for dementia using brain imaging data.

## Figures and Tables

**Table 1:** Simulation results for $e_{0}=$ 0.005, $w_{X}$ = 5, $w_{Y}$ = 20, $w_{u,0}$ = 0.2.

$\mu_{XZ}$ $,\;\mu_{YZ}$	comp1	comp2	Xmean	comp1	comp2	Ymean	XYmean
0.00, 0	0.348	0.351	0.350	**0.021**	0.028	**0.024**	0.187
0.00, 0.25	0.356	0.358	0.357	0.020	0.027	0.023	0.190
0.00, 0.5	0.355	0.311	0.333	0.019	0.028	0.023	0.178
0.00, 0.75	0.364	0.283	0.324	0.021	0.023	0.022	0.173
0.25, 0	0.349	0.287	0.318	0.018	0.026	0.022	0.170
0.25, 0.25	0.349	0.280	0.314	0.018	0.023	0.021	0.168
0.25, 0.5	0.337	0.283	0.310	0.019	0.026	0.022	0.166
0.25, 0.75	**0.381**	**0.389**	**0.385**	0.020	0.013	0.017	**0.201**
0.50, 0	0.348	0.294	0.321	0.017	**0.028**	0.022	0.172
0.50, 0.25	0.350	0.292	0.321	0.017	0.021	0.019	0.170
0.50, 0.5	0.381	0.389	0.385	0.020	0.013	0.017	0.201
0.75, 0	0.348	0.280	0.314	0.018	0.028	0.023	0.168
0.75, 0.25	0.381	0.389	0.385	0.020	0.013	0.017	0.201

**Table 2:** Simulation results for $e_{0}=$ 0.005, $w_{X}$ = 5, $w_{Y}$ = 1, $w_{u,0}$ = 0.8.

$\mu_{XZ}$ $,\;\mu_{YZ}$	comp1	comp2	Xmean	comp1	comp2	Ymean	XYmean
0.00, 0	**0.510**	0.870	**0.690**	0.466	0.690	0.578	0.634
0.00, 0.25	0.504	0.862	0.683	0.463	0.690	0.577	0.630
0.00, 0.5	0.504	0.853	0.678	0.470	0.693	0.581	0.630
0.00, 0.75	0.499	0.853	0.676	**0.473**	0.686	0.580	0.628
0.25, 0	0.506	0.863	0.684	0.464	0.705	0.584	0.634
0.25, 0.25	0.499	0.864	0.681	0.460	0.704	0.582	0.632
0.25, 0.5	0.493	0.874	0.684	0.472	**0.708**	**0.590**	0.637
0.25, 0.75	0.375	0.602	0.489	0.362	0.008	0.185	0.337
0.50, 0	0.506	0.863	0.685	0.465	0.704	0.584	0.634
0.50, 0.25	0.489	0.868	0.679	0.457	0.701	0.579	0.629
0.50, 0.5	0.375	0.602	0.489	0.362	0.008	0.185	0.337
0.75, 0	0.505	**0.874**	0.690	0.465	0.704	0.584	**0.637**
0.75, 0.25	0.375	0.602	0.489	0.362	0.008	0.185	0.337

## Data Availability

The data utilized in this study were obtained from the Alzheimer’s Disease Neuroimaging Initiative (ADNI) database (adni.loni.usc.edu).

## Appendix A

Table A1 and Table A2 list additional results for other parameter-setting patterns in the simulation study.

**Table A1:** Simulation results for $e_{0}$ = 0.005, $w_{X}$ = 5, $w_{Y}$ = 1, $w_{u,0}$ = 0.2.

$\mu_{XZ}$ $,\;\mu_{YZ}$	comp1	comp2	Xmean	comp1	comp2	Ymean	XYmean
0.00, 0	0.502	0.844	0.673	0.432	0.140	0.286	0.480
0.00, 0.25	0.504	0.829	0.666	0.439	0.139	0.289	0.478
0.00, 0.5	0.501	0.841	0.671	**0.440**	0.136	0.288	0.479
0.00, 0.75	0.504	0.800	0.652	0.437	0.132	0.284	0.468
0.25, 0	0.506	**0.848**	**0.677**	0.437	0.139	0.288	**0.482**
0.25, 0.25	0.503	0.841	0.672	0.439	0.136	0.288	0.480
0.25, 0.5	0.502	0.818	0.660	0.435	0.130	0.282	0.471
0.25, 0.75	0.336	0.634	0.485	0.297	−0.024	0.137	0.311
0.50, 0	0.505	0.842	0.674	0.433	0.139	0.286	0.480
0.50, 0.25	0.503	0.829	0.666	0.436	0.139	0.287	0.477
0.50, 0.5	0.336	0.634	0.485	0.297	−0.024	0.137	0.311
0.75, 0	**0.506**	0.840	0.673	0.439	**0.140**	**0.289**	0.481
0.75, 0.25	0.336	0.634	0.485	0.297	−0.024	0.137	0.311

**Table A2:** Simulation results for $e_{0}$ = 0.005, $w_{X}$ = 5, $w_{Y}$ = 20, $w_{u,0}$ = 0.8.

$\mu_{XZ}$ $,\;\mu_{YZ}$	comp1	comp2	Xmean	comp1	comp2	Ymean	XYmean
0.00, 0	0.344	0.383	0.364	0.021	0.026	0.024	0.194
0.00, 0.25	0.367	0.366	0.367	0.018	0.028	0.023	0.195
0.00, 0.5	0.343	0.360	0.352	0.019	0.030	0.024	0.188
0.00, 0.75	0.335	0.344	0.339	**0.022**	0.029	**0.025**	0.182
0.25, 0	0.354	0.304	0.329	0.019	0.030	0.024	0.177
0.25, 0.25	0.354	0.313	0.333	0.019	0.026	0.022	0.178
0.25, 0.5	0.346	0.330	0.338	0.018	0.029	0.024	0.181
0.25, 0.75	**0.383**	**0.394**	**0.388**	0.020	0.015	0.018	**0.203**
0.50, 0	0.343	0.295	0.319	0.017	0.031	0.024	0.171
0.50, 0.25	0.356	0.300	0.328	0.016	0.028	0.022	0.175
0.50, 0.5	0.383	0.394	0.388	0.020	0.015	0.018	0.203
0.75, 0	0.346	0.307	0.327	0.017	**0.032**	0.024	0.176
0.75, 0.25	0.383	0.394	0.388	0.020	0.015	0.018	0.203
